# Impact of Deceased Donor Kidney Procurement Biopsy Technique on Histologic Accuracy

**DOI:** 10.1016/j.ekir.2020.08.004

**Published:** 2020-08-14

**Authors:** S. Ali Husain, Vaqar Shah, Hector Alvarado Verduzco, Kristen L. King, Corey Brennan, Ibrahim Batal, Shana M. Coley, Isaac E. Hall, M. Barry Stokes, Geoffrey K. Dube, R. John Crew, Adler Perotte, Karthik Natarajan, Dustin Carpenter, P. Rodrigo Sandoval, Dominick Santoriello, Vivette D’Agati, David J. Cohen, Lloyd Ratner, Glen Markowitz, Sumit Mohan

**Affiliations:** 1 Department of Medicine, Division of Nephrology, Columbia University College of Physicians and Surgeons and New York Presbyterian Hospital, New York, New York, USA; 2 The Columbia University Renal Epidemiology (CURE) Group, New York, New York, USA; 3 Department of Pathology and Cell Biology, Columbia University College of Physicians and Surgeons and New York Presbyterian Hospital, New York, New York, USA; 4 Department of Medicine, Division of Nephrology and Hypertension, University of Utah School of Medicine, Salt Lake City, Utah, USA; 5 Department of Biomedical Informatics, Columbia University, New York, New York, USA; 6 Department of Surgery, Columbia University College of Physicians & Surgeons and New York Presbyterian Hospital, New York, New York, USA; 7 Department of Epidemiology, Mailman School of Public Health, Columbia University, New York, New York, USA

**Keywords:** deceased donor kidney transplant, epidemiology, kidney procurement biopsy, kidney transplant outcomes, organ quality, organ utilization

## Abstract

**Introduction:**

The factors that influence deceased donor kidney procurement biopsy reliability are not well established. We examined the impact of biopsy technique and pathologist training on procurement biopsy accuracy.

**Methods:**

We retrospectively identified all deceased donor kidney-only transplants at our center from 2006 to 2016 with both procurement and reperfusion biopsies performed and information available on procurement biopsy technique and pathologist (n = 392). Biopsies were scored using a previously validated system, classifying “suboptimal” histology as the presence of at least 1 of the following: glomerulosclerosis ≥11%, moderate/severe interstitial fibrosis/tubular atrophy, or moderate/severe vascular disease. We calculated relative risk ratios (RRR) to determine the influence of technique (core vs. wedge) and pathologist (renal vs. nonrenal) on concordance between procurement and reperfusion biopsy histologic classification.

**Results:**

A total of 171 (44%) procurement biopsies used wedge technique, and 221 (56%) used core technique. Results of only 36 biopsies (9%) were interpreted by renal pathologists. Correlation between procurement and reperfusion glomerulosclerosis was poor for both wedge (*r*^2^ = 0.11) and core (*r*^2^ = 0.14) biopsies. Overall, 34% of kidneys had discordant classification on procurement versus reperfusion biopsy. Neither biopsy technique nor pathologist training was associated with concordance between procurement and reperfusion histology, but a larger number of sampled glomeruli was associated with a higher likelihood of concordance (adjusted RRR = 1.12 per 10 glomeruli, 95% confidence interval = 1.04−1.22).

**Conclusions:**

Biopsy technique and pathologist training were not associated with procurement biopsy histologic accuracy in this retrospective study. Prospective trials are needed to determine how to optimize procurement biopsy practices.

## Graphical abstract

**Figure:**
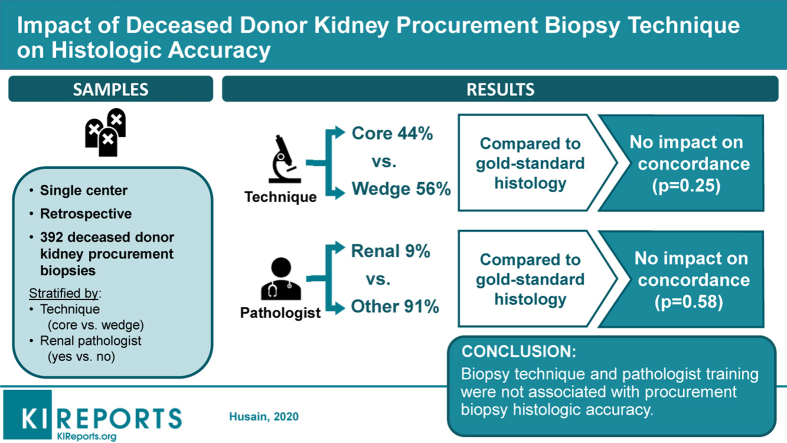

An ongoing shortage of kidneys available for transplantation in the United States has been exacerbated by the suboptimal use of deceased donor kidneys.^1^^,^^2^ One of every 5 recovered deceased donor kidneys is discarded, and only 16% of those that are transplanted are never declined during allocation.^2^, ^3^, ^4^ Organ quality concerns are the predominant reason for reluctance toward kidney use.^4^ In particular, perceived unfavorable findings on procurement biopsies are the most commonly cited justification for kidney discard.^1^^,^^5^

As a result, there has been significant interest in determining the utility of these biopsies.^6^, ^7^, ^8^, ^9^, ^10^ We previously demonstrated that histologic findings of high-quality reperfusion biopsies performed after allograft implantation and interpreted by expert pathologists were associated with post-transplantation allograft outcomes at our center.^11^ However, this relationship between histology and outcomes was present only for a subset of procurement biopsies performed in a standardized fashion by our organ procurement organization and not for procurement biopsies overall.^11^ We therefore hypothesized that 1 or more aspect of how these procurement biopsies are performed and interpreted might have an impact on the accuracy of their histologic findings and explain these discrepant findings. Here, we examine the impact of procurement biopsy technique and pathologist experience on concordance between procurement biopsy findings and reperfusion biopsy findings.

## Materials and Methods

### Study Population

**Figure 1:**
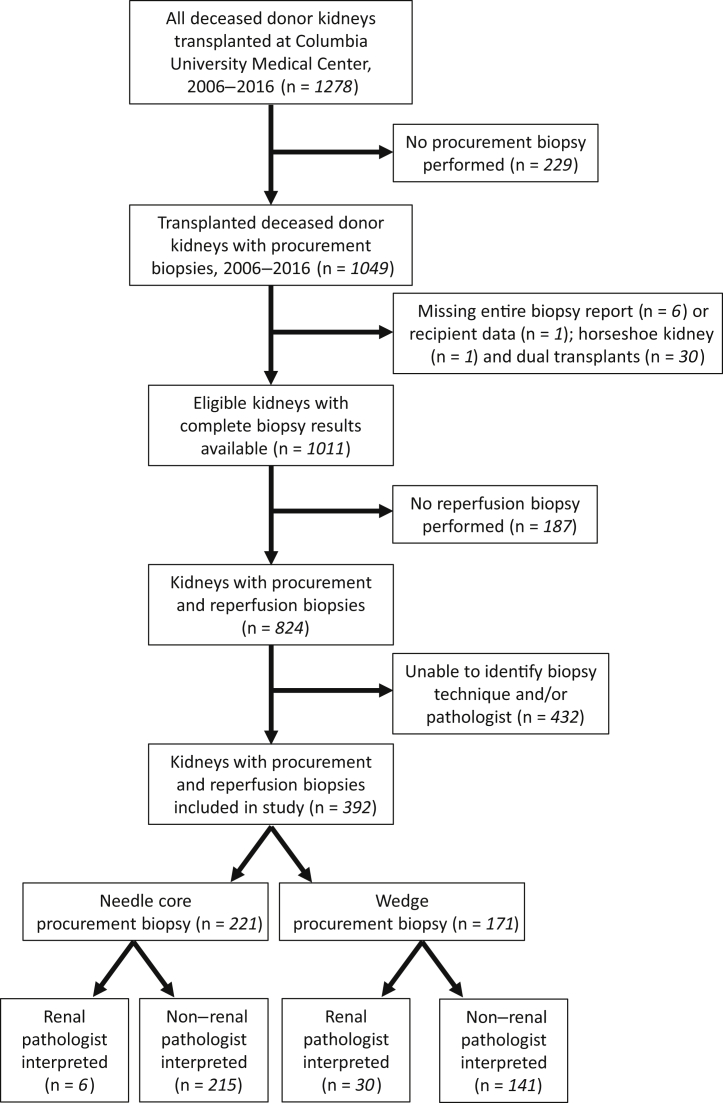
Flow diagram of study cohort.

We identified 1278 deceased donor kidneys transplanted at our center between 1 January 2006 and 31 December 2016 (Figure 1). Of these, 1049 (82%) underwent at least 1 procurement biopsy prior to transplantation. We excluded kidneys used in multiorgan transplants (n=31), as well as those whose biopsy reports or clinical data were missing (n = 7). Of the remaining 1011 kidneys, 824 (82%) also had a reperfusion biopsy performed; the remainder were excluded. In 392 (48%) of these cases, we were able to identify both the procurement biopsy technique used (core vs. wedge) and the pathologist training status (i.e., renal vs. nonrenal pathologist). This study was approved by the Columbia University Medical Center Institutional Review Board. All clinical and research activities associated with this study were consistent with the principles of the Declaration of Istanbul.

### Procurement Biopsy Results and Scoring

Procurement biopsies in the study were frozen section biopsies performed as part of routine clinical care, and the study authors had no role in deciding which kidneys underwent biopsies or which technique was used for any biopsy. All details about biopsy technique and identifying information for pathologists were obtained directly from scanned biopsy reports available in DonorNet. No procurement biopsies were processed or interpreted at our institution.

We compiled information on glomerulosclerosis, interstitial fibrosis and tubular atrophy (IFTA), and vascular disease for each biopsy as reported by the interpreting pathologists. For each of these histologic parameters, we used the previously validated scoring system that assigns a score of 0 (most favorable) to 3 (least favorable) consistent with thresholds from prior studies using this cohort (Table 1).^10^ In cases in which a range of values was reported for any compartment, the lower end of the range was used to assign the histologic score (e.g. “moderate to severe” vascular disease was scored as 2). If more than 1 procurement biopsy was performed, results from the first biopsy were considered in our analysis.

**Table 1:** Histologic scoring for each biopsy compartment

Assigned score	Glomerulosclerosis, %	Interstitial fibrosis and tubular atrophy, %	Vascular disease, %
0	<5	None (0–10)	None (0–10)
1	5–10	Mild (11–25)	Mild (11–25)
2	11–25	Moderate (26–50)	Moderate (26–50)
3	>25	Severe (>50)	Severe (>50)

“Optimal” histology is defined by a score of 0 or 1 for each of the 3 histological parameters, whereas a score of 2 or 3 for at least 1 parameter is designated as “suboptimal.”

“Advanced sclerosis” for a given compartment was defined as a score of ≥2. Overall histologic classification was considered “optimal” for a given kidney in the presence a score of ≤1 for all 3 biopsy compartments (i.e., lack of advanced sclerosis in any compartment). Bilateral concordance was defined as matching “optimal” versus “suboptimal” categorization for both kidneys (right and left) from the same donor. Compartment concordance was considered to be present in a procurement biopsy if there was homogeneity in the presence or absence of advanced sclerosis (i.e., histologic score of ≤1 vs. ≥2) in all 3 components of the biopsy for that kidney.^11^

A total of 12 (3%) procurement biopsy reports did not comment on IFTA, and 19 (5%) were missing information about vascular disease. In these cases, optimal histology was defined by histologic score of ≤1 in all compartments that had recorded data. In cases in which not all compartments were included in the procurement biopsy report, compartment concordance was defined as concordance of all compartments that were recorded.

### Reperfusion Biopsy Results and Scoring

All reperfusion biopsies were also obtained as part of routine clinical care and were evaluated at our institution (Columbia University Irving Medical Center). Two cores of kidney tissue were obtained using an 18-gauge Bard disposable core biopsy needle (Tempe, AZ), formalin fixed, paraffin embedded, and processed using hematoxylin and eosin, periodic acid−Schiff, trichrome, and Jones methenamine silver stains. All biopsies were interpreted by fellowship-trained renal pathologists at our institution whose clinical practice exclusively includes renal pathology, with each pathologist examining more than 600 renal biopsy samples per year. Our previous analyses demonstrated a significant association between findings on these reperfusion biopsies and post-transplantation outcomes in unadjusted and adjusted models.^9^^,^^11^ Reperfusion biopsies were scored using the same scheme as the procurement biopsies (Table 1).

### Classification of Pathologists

Each procurement biopsy pathologist was designated as a “renal pathologist” if at least 1 of the following criteria were met: (i) completed fellowship training in renal pathology as discoverable by online search (8 pathologists, representing 14 biopsies); (ii) website profile listed expertise in renal pathology (an additional 4 pathologists, representing 4 biopsies); and (iii) membership in the Renal Pathology Society at the time of study data collection (January 2020) (an additional 4 pathologists, representing 18 biopsies).

### Donor and Recipient Variables

Donor clinical and demographic data were obtained from the medical record. As recommended by the Organ Procurement and Transplantation Network, the kidney donor risk index and kidney donor profile index were calculated for each donor using a 2015 scaling factor.^12^

### Statistical Analysis

Descriptive statistics were used to compare donor characteristics and procurement biopsy characteristics for kidney biopsies performed using wedge versus core technique. These 2 groups were also compared with regard to concordance between procurement and reperfusion biopsy findings using χ^2^ tests. Overall histologic classification concordance was defined as matching optimal versus suboptimal designation in procurement and reperfusion biopsies. Concordance in each compartment was defined as matching between procurement and reperfusion biopsies with regard to the presence of advanced sclerosis (score 2 or 3) for that compartment.

The primary outcome of interest was concordance in the overall histologic classification between procurement and reperfusion biopsy. Relative risk ratios were calculated to determine the biopsy factors associated with higher odds of concordance between biopsies in univariate and multivariable models.

All statistical analyses were performed using Stata/MP 15 (StataCorp, College Station, TX). Statistical significance was defined as a 2-sided α < 0.05.

## Results

Among the 392 deceased donor kidneys included, 171 (44%) had wedge and 221 (56%) had needle core procurement biopsies (Figure 1). Only 36 procurement biopsies (9%) were interpreted by a renal pathologist—30 in the wedge biopsy group (18%) and 6 in the core biopsy group (3%) (Table 2). The vast majority (85%) of core biopsies were performed by the local organ procurement organization, whereas all but 1 wedge biopsy were performed at an outside organ procurement organization. Kidneys that underwent wedge biopsy were more likely to be from donors with hypertension, obesity, and higher kidney donor risk index compared to kidneys that underwent core biopsy.

**Table 2:** Characteristics of study cohort, stratified by procurement biopsy technique

Characteristic	All (n = 392)	Wedge biopsy (n = 171, 44%)	Core biopsy (n = 221, 56%)	*P*
Donor characteristic				
Age, yr	44 (12)	46 (11)	42 (13)	<0.001
Female sex	161 (41)	73 (43)	88 (40)	0.57
Hypertension	153 (39)	81 (47)	72 (33)	0.003
Diabetes mellitus	53 (14)	24 (14)	29 (13)	0.79
Obesity	264 (67)	127 (74)	137 (62)	0.01
Final creatinine (mg/dl)	2.10 ± 1.87	2.29 ± 1.72	1.95 ± 1.96	0.07
Kidney donor risk index	1.15 ± 0.30	1.21 ± 0.26	1.11 ± 0.33	0.002
Procurement biopsy characteristic				
Renal pathologist	36 (9)	30 (18)	6 (3)	<0.001
Performed at our center’s local organ procurement organization	188 (48)	1 (1)	187 (85)	<0.001
Number of glomeruli	49 ± 30	56 ± 35	44 ± 25	<0.001
Advanced glomerulosclerosis	88 (22)	54 (32)	34 (15)	<0.001
Advanced interstitial fibrosis/tubular atrophy (n = 380)^a^	6/380 (2)	6/165 (4)	0/215 (0)	0.005
Advanced vascular disease (n = 373)^a^	69/373 (18)	19/159 (12)	50/214 (23)	0.005
Compartment concordance	189 (48)	63 (37)	126 (57)	<0.001
Bilateral concordance	283 (72)	139 (81)	144 (65)	<0.001
Comparison to reperfusion biopsy				
Discordant overall histologic classification	134 (34)	62 (36)	72 (33)	0.45
Reperfusion histology more favorable	57 (15)	18 (11)	39 (18)	0.05
Number of discordant compartments				0.03
0	219 (56)	85 (50)	134 (61)	
1	139 (35)	64 (37)	75 (34)	
2	33 (8)	21 (12)	12 (5)	
3	1 (0.3)	1 (0.6)	0 (0)	
Discordant glomerulosclerosis	103 (26)	51 (30)	52 (24)	0.16
Discordant interstitial fibrosis/tubular atrophy (n = 380)^a^	6 (2)	6/165 (4)	0/215 (0)	0.006
Discordant vascular disease (n = 373)^a^	99 (27)	52/159 (33)	47/214 (22)	0.02

Data are n (%) or mean ± SD.

a A total of 12 biopsies were missing information about interstitial fibrosis/tubular atrophy, and 19 were missing information about vascular disease. These biopsies were excluded from the relevant rows.

**Figure 2:**
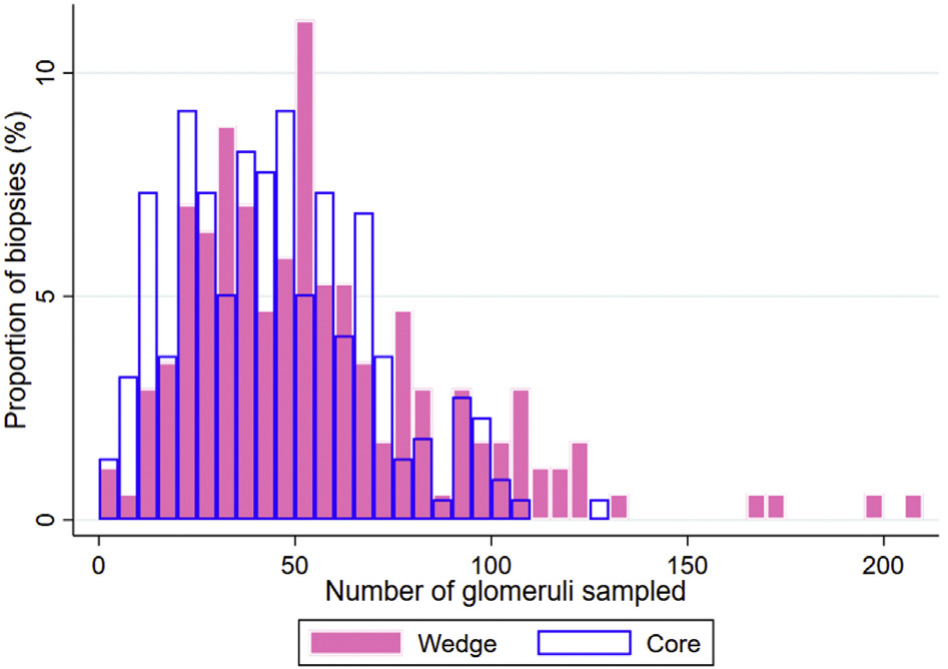
Histogram of number of glomeruli sampled, by procurement biopsy technique. Wedge biopsies were associated with a higher mean number of glomeruli sampled compared to core biopsies (56 ± 35 vs. 44 ± 25, *P* < 0.001).

The wedge biopsy technique was associated with a higher mean number of glomeruli sampled compared to core biopsies (56 ± 35 vs. 44 ± 25, *P* < 0.001) (Figure 2). Wedge biopsies were more likely to be reported to have advanced scarring (defined as a score of 2 or 3) when considering glomerulosclerosis (32% of wedge biopsies vs. 15% of core biopsies, *P* < 0.001) and IFTA (4% vs. 0%, *P* = 0.005), but core biopsies were more likely to be reported to have advanced vascular disease (12% of wedge biopsies vs. 23% of core biopsies, *P* = 0.005) (Table 2).

**Figure 3:**
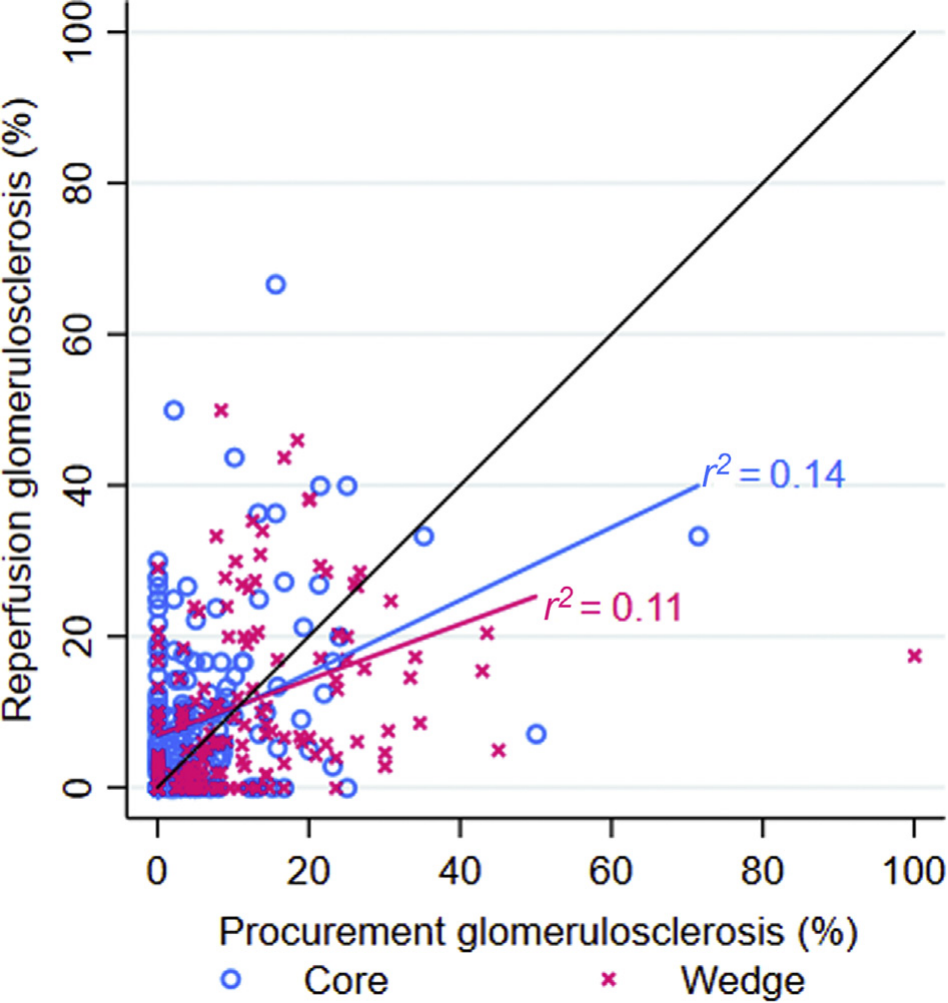
Percent glomerulosclerosis on procurement biopsy versus reperfusion biopsy, by biopsy technique. Correlation between procurement and reperfusion glomerulosclerosis was low regardless of whether wedge (*r*^2^ = 0.11) or core (*r*^2^ = 0.14) technique was used for the procurement biopsy. The black line is a reference for concordance.

A total of 134 kidneys (34%) had discordant optimal versus suboptimal histologic classification on procurement versus reperfusion biopsy, a proportion that was similar among wedge biopsies and core biopsies. However, core biopsies were more likely than wedge biopsies to match the corresponding reperfusion histological biopsy classification for all compartments assessed (61% vs. 50%) (*P* = 0.03) (Table 2). Discordance in the identification of advanced vascular disease on procurement compared to reperfusion histology was higher for wedge biopsies (33%) than for core biopsies (22%) (*P* = 0.02). When treated as a continuous variable, the overall correlation between procurement and reperfusion glomerulosclerosis was poor regardless of biopsy technique (wedge: *r*^2^ = 0.11; core: *r*^2^ = 0.14) (Figure 3).

Finally, we examined the features of procurement biopsies that were associated with concordant overall histologic classification (optimal vs. suboptimal) compared to reperfusion biopsy findings. In univariate and multivariable analyses, neither pathologist training nor biopsy technique was associated with concordance (Table 3). In contrast, a larger number of sampled glomeruli (adjusted relative risk ratio [adjusted RRR] = 1.12 per 10 glomeruli, 95% confidence interval [CI] = 1.04–1.22, *P* = 0.005), bilateral histologic concordance (adjusted RRR = 2.05, 95% CI = 1.26–3.34, *P* = 0.004), and concordance in scoring between compartments for a given kidney (adjusted RRR = 1.73, 95% CI = 1.10–2.73, *P* = 0.018) in a procurement biopsy were all associated with a higher likelihood of concordant histologic classification on reperfusion biopsy.

**Table 3:** Association between procurement biopsy characteristics and risk of concordance between procurement and reperfusion overall histologic classification (i.e., optimal vs. suboptimal)

Characteristic	RRR	95% CI	*P*	Adjusted RRR^a^	95% CI	*P*
Number of glomeruli (per 10)	1.12	1.04–1.20	0.002	1.12	1.04–1.22	0.005
Renal pathologist (vs. nonrenal)	0.70	0.35–1.41	0.32	0.80	0.38–1.72	0.58
Core (vs. wedge)	1.18	0.80–1.73	0.40	1.32	0.82–2.12	0.25
Bilateral concordance on procurement biopsy (yes vs. no)	2.24	1.47–3.40	<0.001	2.05	1.26–3.34	0.004
Compartment concordance on procurement biopsy (yes vs. no)	1.90	1.28–2.81	0.001	1.73	1.10–2.73	0.018

CI, confidence interval; RRR, relative risk ratio.

a The adjusted model only includes all variables listed in this table.

## Discussion

A worsening shortage of deceased donor kidneys available for transplantation, coupled with the current scrutiny of the high kidney discard rate, is expected to increase the use of less-than-ideal kidneys in the United States in the near future.^1^ However, the safe use of these organs requires an accurate and reliable assessment of organ quality. In the United States, there is considerable reliance on procurement biopsies when attempting to ascertain the quality of an organ, and these biopsies are the most commonly cited reason for deceased donor kidney discard.^1^ Given the value of kidney biopsies in clinical management and prognosis for diseases of native and transplanted kidneys, it is not unreasonable for clinicians to expect procurement biopsies to also provide objective and reliable information on deceased donor kidney quality.

However, several previous studies have demonstrated that—unlike high-quality reperfusion biopsies— procurement biopsies are typically poorly reproducible, not reflective of the gold-standard histology to which clinicians are accustomed, and, as a result, poorly predictive of post-transplantation outcomes.^11^, ^12^, ^13^, ^14^, ^15^, ^16^, ^17^ However, although procurement biopsy findings overall are not associated with allograft outcomes, we recently showed that procurement biopsies performed by a specific organ procurement organization are associated with post-transplantation outcomes,^10^ leading to our hypothesis that certain practice pattern variations, such as tissue sampling technique and the prior training of the interpreting pathologist, underlie this finding through their influence on the accuracy of procurement biopsy findings. However, in this study, we found that neither core versus wedge sampling nor the level of renal pathology expertise of the interpreting pathologist was associated with a higher likelihood of accuracy of procurement histology compared to gold-standard reperfusion histology.

We found that core versus wedge biopsy sampling technique had no impact on the likelihood of concordance with gold-standard histology for overall histologic classification. This contradicts our initial hypothesis that core biopsies would more closely reflect gold-standard histology as a result of potential oversampling of scarred subcapsular tissue in a wedge biopsy. Furthermore, because donors whose kidneys underwent wedge biopsy in our cohort were more likely to have had characteristics associated with increased kidney scarring, including older age, hypertension, obesity, and higher creatinine, we cannot directly comment on whether wedge biopsies yield a disproportionate amount of scarred subcapsular tissue overall. Notably, however, core biopsies were more likely to yield concordance between procurement and reperfusion histology on the assessment of all individual histologic compartments. This finding was driven primarily by the higher rate of discordance in the assessment of advanced vascular disease seen with wedge biopsies. Given the similar performance of core and wedge procurement biopsies overall and in the assessment of glomerulosclerosis, the significance of this isolated finding is not clear. Further studies comparing wedge versus core biopsies performed on the same kidneys, processed using the same techniques and read by the same pathologists, are needed to definitively answer whether biopsy technique has a direct and measurable impact on the reliability of kidney biopsy findings.

A larger tissue sample, as measured by the number of glomeruli obtained, was associated with a higher likelihood that procurement histology would be concordant with reperfusion biopsy histology. However, the mean number of glomeruli sampled in our cohort was much higher than the minimum required for a biopsy to be deemed adequate independent of biopsy technique, so the implications of this finding for current procurement biopsy practices is unclear: core procurement biopsies sampled a mean of 44 ± 25 glomeruli, greater that what may be expected of a typical core biopsy performed in clinic practice. Furthermore, we are unable to take into account other factors that influence sample adequacy, such as number and type of blood vessels sampled. As expected, instances in which there was histologic concordance between both kidneys from a given donor, as well as those with concordance in scoring between compartments for a given kidney, resulted in greater agreement between procurement histology and reperfusion histology. These findings also suggest that biopsy specimens with an isolated finding of advanced sclerosis in only 1 compartment or in only 1 kidney from a pair may not be an accurate representation of the overall quality of the organ. Such findings on a procurement biopsy should raise concerns about the validity of the findings and the biopsy result as a whole.^10^ For example, a finding of high glomerulosclerosis but minimal or absent IFTA and vascular disease should prompt suspicion of sampling error. Given the relatively low sample size compared to overall kidney parenchyma inherent in any procurement biopsy, it is not surprising that the impact of increasing glomerular sample size on improving histologic accuracy was modest. This inherent sampling limitation is likely worsened by a lack of standardization around which areas of the kidney should be sampled, in light of evidence from prior studies showing differing assessments of nephrosclerosis when multiple procurement biopsies are performed on different areas of the same kidney.^15^ Even under ideal circumstances, any kidney biopsy, including procurement biopsies, is subject to potential sampling error.

Prior studies have attempted to identify the ideal method for performing procurement biopsies, with inconsistent results. In 1 analysis, serial wedge biopsies were performed on 9 discarded kidneys and found likely overestimation of glomerulosclerosis in subcapsular wedge tissue.^15^ However, no direct comparison to core biopsies was made. Other investigators found core biopsies to sample a greater number of arterioles despite sampling fewer glomeruli than wedge biopsies.^17^ Similarly, a small study of living donor kidneys compared wedge biopsies to subsequent core biopsies and found that the biopsy pairs primarily differed on the assessment of arterial fibrointimal thickening, with only core biopsy results appropriately correlating with age.^14^ In contrast, an additional retrospective study of procurement biopsies from 20 unique donors showed inferior interrater agreement in the assessment of vascular disease and glomerulosclerosis for core biopsies compared to wedge biopsies.^18^ However, our study is unique in its comparison to gold-standard reperfusion histology rather than to additional procurement biopsies.

Only 9% of procurement biopsies in our cohort were interpreted by renal pathologists, limiting our ability to meaningfully assess the impact of prior renal pathology training on procurement biopsy interpretation. With this limitation in mind, we found no association between pathologist expertise and concordance between procurement biopsy histology and reperfusion biopsy histology. In contrast, Azancot *et al.* compared retrospective procurement biopsy review by a trained renal pathologist to biopsy interpretations that had been done by on-call pathologists, and found that only biopsy results from the renal pathologist were associated with subsequent post-transplantation outcomes.^13^ However, the investigators’ reliance on a single renal pathologist, and their use of multiple stains for procurement biopsy assessment (hematoxylin–eosin, periodic acid–Schiff, and Masson’s trichrome) make our results difficult to compare directly. Overall, it is possible that renal pathology training alone is insufficient to reflect the skill level in interpreting procurement biopsies, and that pathologists who lack specific renal training but interpret a large quantity of kidney biopsies can adequately assess nephrosclerosis on procurement biopsies. However, additional data are needed to help organ procurement organizations determine which qualifications they should require of pathologists who read their procurement biopsies.

In light of our findings, it is possible that variability in tissue processing and staining are instead the primary factors that influence procurement biopsy accuracy, whereas variables such as biopsy technique do not have an impact. Although procurement biopsy staining and preparation technique were not explicitly stated in the majority of biopsy reports, almost all procurement biopsies in the United States are frozen section specimens.^19^^,^^20^ This technique can make the evaluation of features such as IFTA difficult because of distortion of tubulointerstitial structures, and some centers recommend against the reporting of IFTA based on these specimens.^21^, ^22^, ^23^ The preparation of formula-fixed, paraffin-embedded specimens is more costly and time consuming, but these downsides must be weighed against the benefits of more accurate histologic assessments.

We should note that, similar to other retrospective procurement biopsy cohorts, our results are likely influenced by selection bias introduced at several points. First, kidneys whose procurement biopsies show perceived extremely poor histologic findings are more likely to be discarded and systematically excluded from analyses like ours, so our ability to study the reliability of those findings is limited. In addition, the decision as to whether or not to biopsy a given deceased donor kidney is based on different criteria at each organ procurement organization, making it difficult to compare biopsy cohorts from different donation service areas with unclear criteria for when a biopsy is performed. Furthermore, individual organ procurement organizations are likely internally consistent in their use of a given biopsy technique and pathologist(s). The interaction between biopsy criteria, technique, and interpretation, as well as other residual confounders (for example, quality of staining and slide preparation), therefore make valid comparison between these factors challenging with selective retrospective data. In addition, a large number of biopsies in our cohort were excluded because of missing information regarding biopsy technique or interpreting pathologist. Our reliance on searchable data about each pathologist to identify renal pathology expertise might also have misclassified renal pathologists whose websites are not updated with their area of expertise or who previously had Renal Pathology Society membership that ended before study data were collected. Finally, useful data on other benefits and risks of different techniques, such as cost, time, equipment required, and associated theoretical bleeding risk, are not available. Overcoming these limitations through the use of a prospective, multicenter study comparing biopsy practices is critical in order to better understand the best role for procurement biopsies in the assessment of kidney quality during organ allocation.

In conclusion, we found no discernible impact of core versus wedge biopsy technique on the concordance between procurement biopsy findings and gold-standard reperfusion biopsy findings for deceased donor kidney allografts. We were unable to meaningfully compare the impact of renal pathology training on procurement biopsy interpretation because of the small number of procurement biopsies interpreted by renal pathologists. Additional prospective studies to determine the factors contributing to the inconsistent accuracy of procurement biopsies are urgently needed to guide efforts to standardize procurement biopsy practice.

## Disclosure

All the authors declared no competing interests.
